# Pulmonary Adenocarcinoma Presenting as a Pineal Gland Mass With Obstructive Hydrocephalus

**DOI:** 10.31486/toj.18.0159

**Published:** 2020

**Authors:** Mohamed A. Abdallah, Mahum Shahid, Moataz Ellithi, Ahmed Yeddi, Arwyn Cunningham, Ryan Askeland, Jamal Dodin

**Affiliations:** ^1^ Department of Internal Medicine, University of South Dakota Sanford School of Medicine, Sioux Falls, SD; ^2^ Department of Internal Medicine, Wayne State University/Detroit Medical Center, Detroit, MI; ^3^ Department of Pathology, University of South Dakota Sanford School of Medicine, Sioux Falls, SD; ^4^ Sanford Health Pathology Clinic, Sioux Falls, SD

**Keywords:** *Adenocarcinoma of lung*, *carcinoma–non small cell lung*, *hydrocephalus*, *neoplasm metastasis*, *pineal gland*

## Abstract

**Background:** Adenocarcinoma is the most prevalent type of non–small cell carcinoma of the lungs. Patients with lung adenocarcinoma often present with cough, dyspnea, pain, and weight loss. They can also present with signs and symptoms of brain metastasis because the lungs are one of the most common origins of metastatic brain cancer. We describe a rare case of adenocarcinoma of the lungs presenting with pineal region metastasis.

**Case Report:** A 61-year-old male presented to the emergency department with dizzy spells and gait disturbance. Magnetic resonance imaging of the brain demonstrated a solitary mass in the pineal region with marked obstructive hydrocephalus. A stereotactic biopsy was performed, and metastatic adenocarcinoma consistent with pulmonary origin was diagnosed. Computed tomography scan of the chest revealed a spiculated mass. The patient died shortly after the diagnosis was made.

**Conclusion:** The pineal region is an unusual site for brain metastasis. Although such metastasis has rarely been described, it should be considered in the differential diagnosis of pineal region tumors, especially for patients with suggestive clinical or histopathologic features of primary malignancy elsewhere.

## INTRODUCTION

Adenocarcinoma of the lungs accounts for nearly 40% of all lung cancers and is the most prevalent type of non–small cell carcinoma of the lung.^1^ These patients commonly present with cough, dyspnea, and weight loss.^2^ Pulmonary carcinomas are the most common primary source of brain metastasis^3^; however, metastasis to the pineal gland is very rare, accounting for only 0.4% of all brain metastases.^1^ We describe a unique presentation of pulmonary adenocarcinoma in a patient presenting with ataxia, presyncopal spells, and falls secondary to pineal metastasis discovered on brain imaging.

## CASE REPORT

A 61-year-old male presented to the emergency department (ED) with a 3-week history of early-morning headaches, presyncopal episodes, blurry vision, and gait disturbance. He also described nausea, dizziness, presyncope, and multiple falls during the same period. The patient denied history of trauma or symptoms of palpitations, weakness, or speech difficulties. The patient's medical history was pertinent for coronary artery disease, chronic obstructive pulmonary disease, peripheral vascular disease, hypertension, and nicotine use disorder. He was an active cigarette smoker with an 84 pack-year smoking history but no alcohol use. His home medications included albuterol, atorvastatin, umeclidinium-vilanterol, pantoprazole, aspirin, and clopidogrel. Vital signs were normal. Physical examination was notable for a bilateral sixth nerve palsy with bilateral papilledema, mild ataxia of both lower extremities accompanied by truncal ataxia, and hyperreflexia in both upper and lower extremities with upgoing plantar reflexes bilaterally. The patient was alert and oriented, could follow commands, and had no aphasia or dysarthria. His pupils were equal, round, and reactive to light bilaterally.

**Figure 1.**
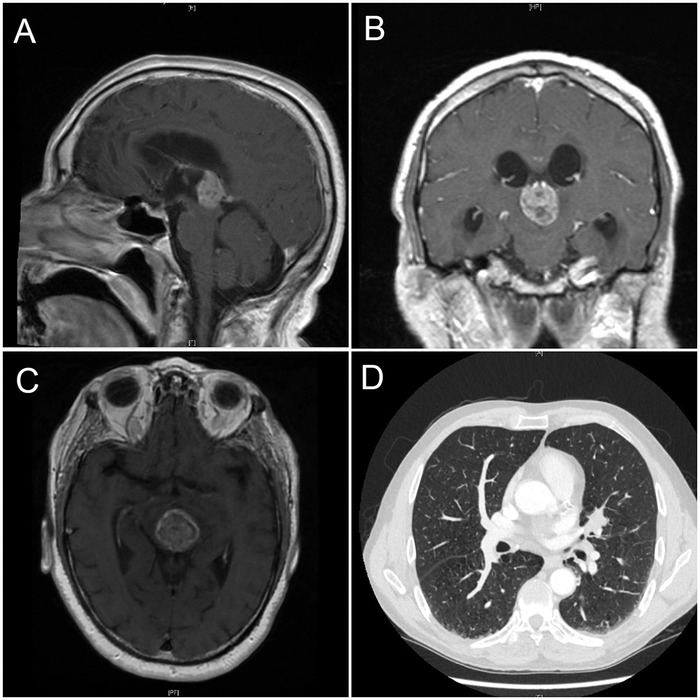
**(A) Sagittal sections of magnetic resonance imaging (MRI) of the brain showing the pineal gland mass. (B) Coronal and (C) transverse sections of brain MRI showing the same pineal tumor. (D) Computed tomography of the chest with intravenous contrast showing a lung nodule over the left hilar region in the lower right corner.**

Head computed tomography (CT) scan performed in the ED showed a soft tissue mass in the pineal gland region with lateral and third ventricular enlargement. Brain magnetic resonance imaging (MRI) showed a 2.1 cm × 2.2 cm pineal mass with marked hydrocephalus from compression of the aqueduct and associated vasogenic brain edema (Figures 1A, 1B, and 1C). CT imaging of the chest, abdomen, and pelvis with intravenous (IV) contrast showed a 2.0 cm × 2.0 cm left hilar spiculated nodule with adjacent lymphadenopathy concerning for malignancy (Figure 1D).

The neurosurgery service evaluated the patient and recommended brain biopsy. The patient requested a second opinion and was transferred to a referral center with neurosurgical expertise. At that center, the patient underwent endoscopic ventriculostomy to relieve hydrocephalus and pineal mass region biopsy. Histologic examination was suggestive of an epithelioid neoplasm with low proliferative activity. The immunophenotype of the tumor was positive for cytokeratin CAM 5.2, cytokeratin OSCAR, and epithelial membrane antigen (EMA), and negative for synaptophysin, glial fibrillary acidic protein (GFAP), neurofilament, S100, and OCT 3/4. Differential diagnosis was low-grade neoplasm such as papillary tumor of the pineal region (PTPR) based on immunoreactivity for cytokeratins and negativity for GFAP and synaptophysin.

Postoperatively, the patient's gait and headaches markedly improved, but he had persistent diplopia. Alternate eye patching was recommended. The patient was discharged with plans for repeat outpatient brain imaging to monitor the size of the tumor given the negative initial brain biopsy.

Four weeks later, the patient presented to the ED after a fall. He was found to have a right hip fracture. The patient reported that shortly after his brain biopsy, his neurologic symptoms had worsened. He required a walker to ambulate and fell more often because of imbalance and ataxia. He continued to have headaches, dizziness, and double vision. MRI of the brain in the ED showed an increase in mass size from 2.1 cm × 2.2 cm to 3.8 cm × 3.3 cm with vasogenic edema around the pineal mass. A decision to proceed with repeat stereotactic biopsy of the pineal tumor was made. Postoperatively, the patient failed to wake up from anesthesia. He had large pupils on neurologic examination. He was transferred to the neurointensive care unit for close monitoring and mechanical ventilation. Repeat CT scan of the head revealed hemorrhage within the mass, as well as small intraventricular hemorrhage. Serial CT head scans demonstrated progressive enlargement of the lateral ventricles. An emergent external ventricular drain was inserted; however, the patient did not show any clinical improvement.

**Figure 2.**
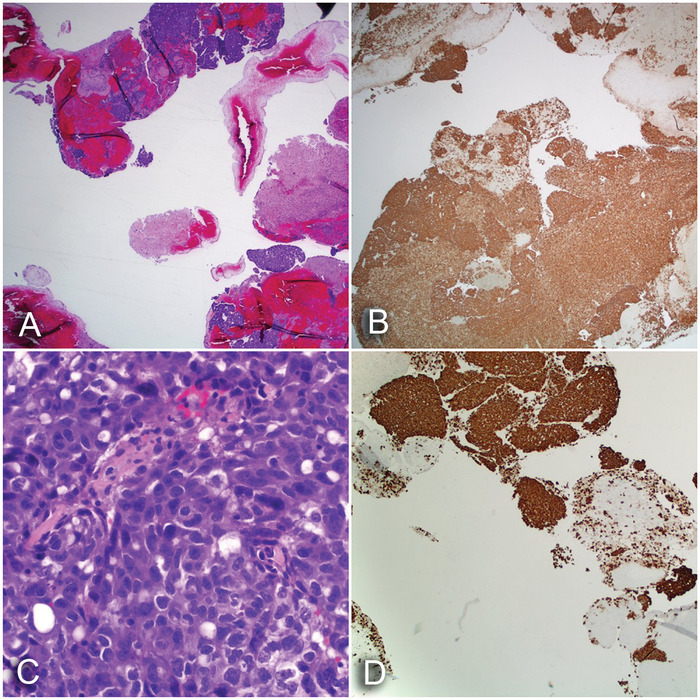
**(A) Epithelioid neoplasm (hematoxylin and eosin [H&E] stain, ×20). (B) Strong and diffuse staining for pankeratin (H&E stain, ×20). (C) Epithelioid neoplasm demonstrating marked nuclear pleomorphism with eosinophilic cytoplasm (H&E stain, ×400). (D) Strong and diffuse staining of the neoplasm for thyroid transcription factor-1, supporting the diagnosis of adenocarcinoma of pulmonary origin (H&E stain, ×40).**

Results of the second biopsy demonstrated fragments of solid tumor intermixed with brain parenchyma and blood. The neoplastic cells had eosinophilic cytoplasm, marked nuclear pleomorphism with irregular nuclear contours, conspicuous nucleoli, and occasional atypical mitoses. The neoplastic cells stained positive for pankeratin, CK7, and thyroid transcription factor-1 (TTF-1). The neoplastic cells were negative for CK20, p40, placental alkaline phosphatase, S100, GFAP, chromogranin A, and synaptophysin. Given the positivity of the CK7 and TTF-1 immunohistochemical stains, the tumor was diagnosed as metastatic adenocarcinoma consistent with pulmonary origin (Figure 2).

The patient remained comatose for 48 hours after the procedure. After discussion with the family about treatment options and prognosis, the family opted for comfort care. The patient was extubated and died shortly thereafter.

## DISCUSSION

Pineal metastases from lung cancer are extremely rare. Patients with pineal metastases are typically asymptomatic, and the cancer is found incidentally on autopsy in most patients.^1^ Small cell carcinoma is the most reported lung cancer associated with pineal metastasis.^1^ Other histologic types, including squamous cell carcinoma^4^ and adenocarcinoma,^1^ have also been reported. Our case describes an atypical presentation of lung adenocarcinoma with hydrocephalus caused by mass effect of metastasis to the pineal gland. In addition, the initial pineal gland biopsy had a broad differential, including a PTPR secondary to the nonspecific staining pattern of the small tumor fragments, with immunoreactivity for cytokeratins and negativity for GFAP and synaptophysin.^5^ PTPR is a rare entity itself, and in 2007, the World Health Organization described PTPR as “a rare neuroepithelial tumor of the pineal region in adults, characterized by papillary architecture and epithelial cytology, immunopositivity for cytokeratin and ultra-structural features suggesting ependymal differentiation.”^6^

Looking retrospectively, the markers in our patient might have suggested a metastatic etiology initially, considering EMA positivity and GFAP negativity.^5^ However, the rarity of both tumor types—primary pineal tumors and metastatic carcinoma—in this region made reaching the correct diagnosis challenging.

Our patient had an isolated brain metastasis to the pineal region, and the unremarkable features of the first pineal biopsy might have obscured the clinical picture initially, leading to a delay in diagnosis. However, multiple biopsies from the same mass can clarify the diagnosis in cases of rare tumors, as the second biopsy in our case did yield a diagnosis.

Another important consideration is the aggressive nature of the adenocarcinoma in this patient, manifested by the increase in size of the pineal mass during the 4-week interval after the first presentation. The rapid progression of this tumor demonstrates that the time window can be very narrow from the time of presentation to initiation of treatment with such malignancies. Because the incidence of primary pineal tumors is low in older patients, any mass in the pineal gland region should always prompt a detailed investigation to look for any primary malignancy, as both types of tumors have drastically different treatment modalities. Because the lungs are the most common primary site for brain metastasis, followed by breast cancer and malignant melanoma,^1^ the index of suspicion should always be high for a possible pineal metastasis, and workup should be done to look for a primary source.

In cases of metastatic malignancy to the brain, after examination of the histology on hematoxylin and eosin stained slides, immunohistochemical stains may be used to help elucidate the site of origin. In metastatic nonmucinous pulmonary adenocarcinoma, TTF-1 is typically positive in >85% of tumor cells. Also helpful in the diagnosis of pulmonary adenocarcinoma is positivity of napsin A and CK7, with negative staining of CK20.^7^

## CONCLUSION

The pineal gland is an extremely rare site for both primary brain tumor and metastatic cancers. In patients with high suspicion for malignant pineal gland tumor, repeat pineal biopsy might be required if the first biopsy is not consistent with the overall clinical picture. As lungs are the most common primary site for brain metastasis, chest imaging and immunohistochemical staining are useful tools for diagnosis of metastatic lung cancer to the pineal gland.

## Notes

This article meets the Accreditation Council for Graduate Medical Education and the American Board of Medical Specialties Maintenance of Certification competencies for Patient Care and Medical Knowledge.
